# 306. Determining natural killer cell responses to JC polyomavirus

**DOI:** 10.1093/ofid/ofad500.378

**Published:** 2023-11-27

**Authors:** C Sabrina Tan, Jenny Ahn, Sarah Chen, Esther Lee, Eunice Gikundiro, Melissa Craemer, Simon Gressens, Taylor Yoder, Michelle Lifton, Stephanie Jost

**Affiliations:** University of Iowa, Iowa City, Iowa; Beth Israel Deaconess Medical Center, Boston, Massachusetts; Beth Israel Deaconess Medical Center, Boston, Massachusetts; Duke University, Durham, North Carolina; University of Iowa, Iowa City, Iowa; Duke University, Durham, North Carolina; Duke University, Durham, North Carolina; Duke University, Durham, North Carolina; Beth Israel Deaconess Medical Center, Boston, Massachusetts; Duke University, Durham, North Carolina

## Abstract

**Background:**

JC polyomavirus (JCPyV) infects oligodendrocytes and causes progressive multifocal leukoencephalopathy (PML), a potentially fatal complication of monoclonal antibody treatments for cancer and autoimmune diseases, and a rare but often-fatal disease in people living with HIV. There is currently no effective treatment against JCPyV and novel immunotherapies for PML are urgently needed. Natural killer (NK) cells play critical roles in defense against viral infections, yet NK cell responses to JCPyV remain largely unexplored.

**Methods:**

Intracellular cytokine staining (ICS) of NK and T cells stimulated with overlapping peptide pools covering the JCPyV capsid VP1 was performed. A novel flow cytometry-based assay was created to determine NK cell killing efficiency of infected SVG-A cells. Blocking antibodies were used to determine NK cell receptors responsible for immune recognition of JCPyV-infected cells. Single cell cloning was used to expand a homogenous population of NK cells.

**Results:**

Using ICS, about 40% of healthy donors demonstrated NK cell responses, with CD107a upregulation and robust IFNy production by NK cells extending beyond T cell responses(Fig 1). Next, we showed that co-culture of NK cells and JCPyV-infected SVG-A cells leads to, on average, a 65% reduction of infected cells (Fig2). Expression of ligands for the activating NK cell receptor NKG2D was modulated in JCPyV-infected cells, with overall enhanced expression of ULBP-2. Accordingly, NKG2D blockade resulted in decreased NK cell degranulation. Interestingly, we also identified JCPyV-derived peptides that elicit dominant responses by NK cells and can stabilize HLA-E, the ligand for the inhibitory NKG2A and the activating NKG2C receptors. Blockade of NKG2A enhanced NK cell response to JCPyV-infected targets. Finally, we have isolated and expanded single NK cell clones with potent cytotoxic functions against autologous target cells pulsed with JCPyV-derived peptides (Fig 3) and JCPyV-infected SVG-A cells.

**Figure:**
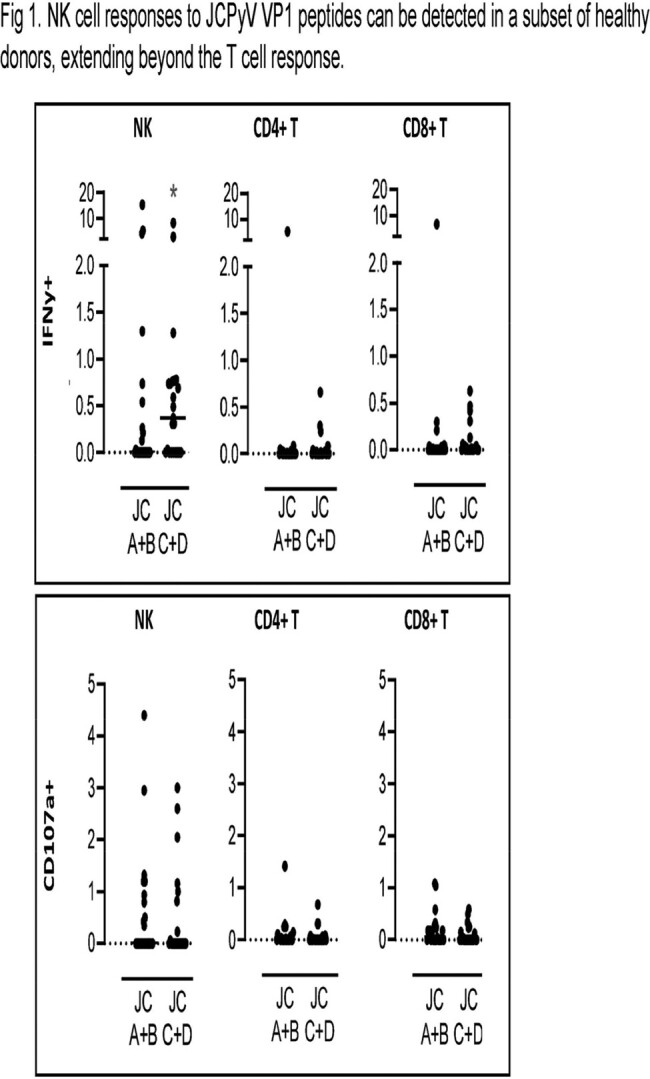

**Fig 2.**
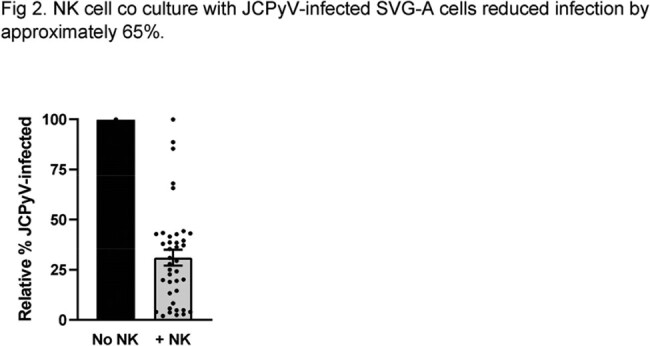

**Fig. 3:**
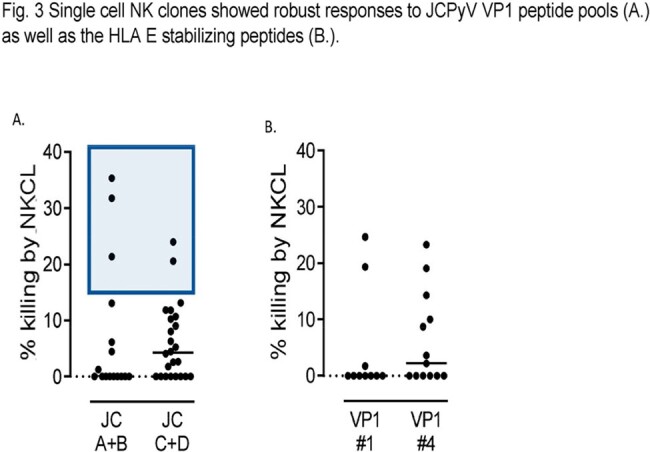

**Conclusion:**

Altogether, these findings suggest NKG2D-mediated activation and the NKG2/HLA-E axis may play key roles in controlling JCPyV replication and targeting these receptors and/or expansion of JCPyV-specific NK cells may be promising NK cell-based immunotherapeutic for PML.

**Disclosures:**

**All Authors**: No reported disclosures

